# Update: Influenza Activity — United States, September 28–December 6, 2014

**Published:** 2014-12-19

**Authors:** Melissa Rolfes, Lenee Blanton, Lynnette Brammer, Sophie Smith, Desiree Mustaquim, Craig Steffens, Jessica Cohen, Michelle Leon, Sandra S. Chaves, Anwar Isa Abd Elal, Larisa Gubareva, Henrietta Hall, Teresa Wallis, Julie Villanueva, Xiyan Xu, Joseph Bresee, Nancy Cox, Lyn Finelli

**Affiliations:** 1 Influenza Division, National Center for Immunization and Respiratory Diseases, CDC

CDC collects, compiles, and analyzes data on influenza activity year-round in the United States (http://www.cdc.gov/flu/weekly/fluactivitysurv.htm). The influenza season generally begins in the fall and continues through the winter and spring months; however, the timing and severity of circulating influenza viruses can vary by geographic location and season. Influenza activity in the United States increased starting mid-October through December. This report summarizes U.S. influenza activity* during September 28–December 6, 2014.†

## Viral Surveillance

**FIGURE 1:**
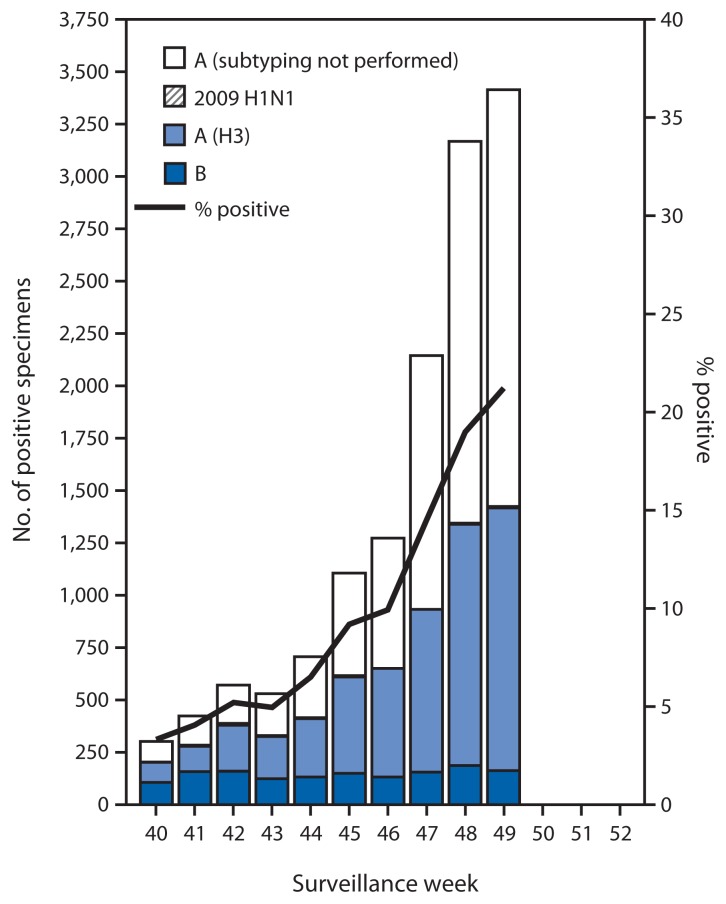
Number* and percentage of respiratory specimens testing positive for influenza, by type, surveillance week, and year — U.S. World Health Organization and National Respiratory and Enteric Virus Surveillance System collaborating laboratories, United States, 2014–15 influenza season^†^ * N = 13,641. ^†^ Data reported as of December 12, 2014.

During September 28–December 6, approximately 250 World Health Organization (WHO) and National Respiratory and Enteric Virus Surveillance System collaborating laboratories in the United States tested 124,618 respiratory specimens for influenza viruses; 13,641 (10.9%) were positive (Figure 1). Of these, 12,175 (89.3%) were influenza A viruses, and 1,466 (10.7%) were influenza B viruses. Of the 12,175 influenza A viruses, 5,122 (42.1%) were subtyped; 5,077 (99.1%) of these were influenza A (H3) viruses, and 45 (0.9%) were influenza A (H1N1)pdm09 (pH1N1) viruses. Since September 28, influenza-positive tests have been reported from 50 states, the District of Columbia, Guam, and Puerto Rico, representing all 10 U.S. Department of Health and Human Services (HHS) regions.§ Thus far, influenza A viruses have predominated nationally and in all 10 HHS regions.

## Influenza Virus Characterization

WHO collaborating laboratories in the United States are requested to submit a subset of their influenza-positive respiratory specimens to CDC for further virus characterization (1). Since October 1, CDC has antigenically or genetically characterized¶ 236 influenza viruses or specimens collected by U.S. laboratories during the 2014–15 season, including 10 pH1N1 viruses, 197 influenza A (H3N2) viruses, and 29 influenza B viruses. All pH1N1 viruses were antigenically like the 2014–15 Northern Hemisphere influenza A vaccine component (A/California/7/2009-like [H1N1]). Of the 197 influenza A (H3N2) viruses, 64 (32.5%) were characterized as A/Texas/50/2012-like (the influenza A [H3N2] component of the 2014–15 Northern Hemisphere influenza vaccine), and 133 (67.5%) showed either reduced titers with antiserum produced against A/Texas/50/2012 or belonged to a genetic group that typically shows reduced titers to A/Texas/50/2012. Among viruses that showed reduced titers with antiserum raised against A/Texas/50/2012, most were antigenically similar to A/Switzerland/9715293/2013, the H3N2 virus selected for the 2015 Southern Hemisphere influenza vaccine. A/Switzerland/9715293/2013 is related to, but antigenically and genetically distinguishable, from the A/Texas/50/2012 vaccine virus. A/Switzerland-like H3N2 viruses were first detected in the United States in small numbers in March of 2014 and began to circulate in greater numbers over the spring and summer. Twenty (69%) of the influenza B viruses tested belong to the B/Yamagata lineage and were characterized as B/Massachusetts/2/2012-like, which is included as an influenza B component in the 2014–15 Northern Hemisphere trivalent and quadrivalent influenza vaccines. The remaining nine (31%) influenza B viruses tested belong to the B/Victoria lineage, and of these, seven (78%) were characterized as B/Brisbane/60/2008-like, which is included as an influenza B component in the 2014–15 Northern Hemisphere quadrivalent influenza vaccine. Two (22%) of the B/Victoria-lineage viruses tested showed reduced titers to B/Brisbane/60/2008.

## Novel Influenza A Viruses

One human infection with an influenza A (H3N2) variant virus (H3N2v) was reported to CDC from Wisconsin during the week ending October 18 (week 42). Contact between the patient and swine in the week preceding illness was reported. The patient was not hospitalized and fully recovered. This is the first H3N2v infection reported for the 2014–15 influenza season.

## Antiviral Resistance of Influenza Viruses

Testing of pH1N1, influenza A (H3N2), and influenza B virus isolates for resistance to neuraminidase inhibitors (oseltamivir and zanamivir) is performed at CDC using a functional assay. Additionally, pH1N1 and influenza A (H3N2) clinical samples are tested for mutations of the virus known to confer oseltamivir resistance. Since October 1, a total of 139 influenza viruses have been assessed for antiviral resistance, including five pH1N1 viruses, 106 influenza A (H3N2) viruses, and 28 influenza B viruses. Of the 139 influenza A and B viruses tested, all were sensitive both to oseltamivir and zanamivir.

## Outpatient Illness Surveillance

**FIGURE 2:**
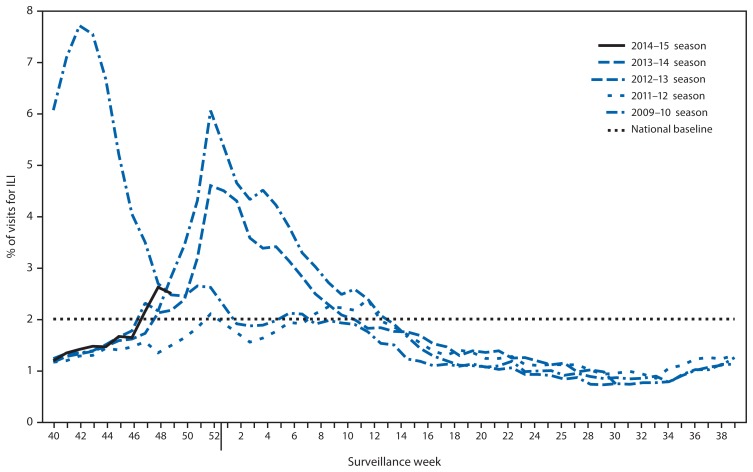
Percentage of all outpatient visits for influenza-like illness (ILI)* reported to CDC, by surveillance week — Outpatient Influenza-Like Illness Surveillance Network, United States, September 28–December 6, 2014, and selected previous influenza seasons^†^ * Defined as a fever (≥100°F [≥37.8°C]), oral or equivalent, and cough and/or sore throat, without a known cause other than influenza. ^†^ Data reported as of December 12, 2014.

Since September 28, the weekly percentage of outpatient visits for influenza-like illness (ILI)** reported by approximately 1,800 U.S. Outpatient ILI Surveillance Network (ILINet) providers in 50 states, New York City, Chicago, the U.S. Virgin Islands, Puerto Rico, and the District of Columbia, which comprise ILINet, has ranged from 1.2% to 2.6% and was first reported to be at or above the national baseline†† of 2.0% during week 47 (week ending November 22) (Figure 2). Peak weekly percentages of outpatient visits for ILI ranged from 2.4% to 7.6% from the 1997–98 through 2013–14 seasons, excluding the 2009 pandemic. Data collected in ILINet are used to produce a measure of ILI activity§§ by jurisdiction. During week 49, Alabama, Georgia, Illinois, Louisiana, Mississippi, Texas, and Puerto Rico experienced high ILI activity, two states (Florida and Indiana) experienced moderate ILI activity, and seven states (Idaho, Kansas, Maryland, Missouri, South Carolina, Utah, and Virginia) experienced low ILI activity. New York City and 35 states experienced minimal ILI activity, and data were insufficient to calculate an ILI activity level for the District of Columbia.

## Geographic Spread of Influenza Activity

For the week ending December 6 (week 49), 14 states (Colorado, Delaware, Florida, Georgia, Illinois, Kentucky, Louisiana, Maryland, Minnesota, New York, North Carolina, Ohio, Pennsylvania, and Texas) reported widespread geographic spread of influenza¶¶, Puerto Rico, Guam, and 25 states (Alabama, Alaska, Arkansas, Connecticut, Indiana, Iowa, Kansas, Maine, Massachusetts, Michigan, Mississippi, Missouri, Montana, Nevada, North Dakota, Oklahoma, Rhode Island, South Carolina, Tennessee, Utah, Vermont, Virginia, Washington, West Virginia, and Wisconsin) reported regional spread, and the U.S. Virgin Islands and seven states reported local spread (Arizona, Idaho, Nebraska, New Hampshire, New Jersey, New Mexico, and Oregon). Sporadic influenza activity was reported by the District of Columbia and four states.

## Influenza-Associated Hospitalizations

CDC monitors hospitalizations associated with laboratory-confirmed influenza in adults and children through the Influenza Hospitalization Surveillance Network (FluSurv-NET),*** which covers approximately 27 million persons, 9% of the U.S. population. From October 1 through December 6 (week 49), 1,028 laboratory-confirmed influenza-associated hospitalizations were reported, yielding a rate of 3.8 per 100,000 population. The highest rate of hospitalization was among adults aged ≥65 years (13.4 per 100,000 population) and young children 0–4 years (6.2 per 100,000 population). Among all hospitalizations, 952 (92.6%) were influenza A, 68 (6.6%) were influenza B, four (0.4%) were influenza A and influenza B coinfections, and four (0.4%) had no virus type information. Among those with influenza A subtype information, 274 (100%) were influenza A (H3N2) viruses.

## Pneumonia- and Influenza-Associated Mortality

During the week ending December 6 (week 49), pneumonia and influenza (P&I) was reported as an underlying or contributing cause of 6.0% (794 of 13,261) of all deaths reported to the 122 Cities Mortality Reporting System. This percentage is below the epidemic threshold of 6.6% for the week.††† Since September 28, the weekly percentage of deaths attributed to P&I ranged from 5.0% to 6.0% and has not exceeded the epidemic threshold so far this season. Peak weekly percentages of deaths attributable to P&I in the previous five seasons ranged from 7.9% during the 2008–09 and 2011–12 seasons to 9.9% during the 2012–13 season.

What is already known on this topic?

CDC collects, compiles, and analyzes data on influenza activity year-round in the United States. The influenza season generally begins in the fall and continues through the winter and spring months; however, the timing and severity of circulating influenza viruses can vary by geographic location and season.

What is added by this report?

During September 28–December 6, 2014, influenza activity overall in the United States has been increasing. Influenza A (H3N2) viruses were the most frequently identified viruses. More than half of the influenza A (H3N2) viruses characterized thus far this season have evidence of reduced reactivity to sera produced against the A/Texas/50/2012-like (H3N2) vaccine virus, a component of the 2014–15 Northern Hemisphere trivalent and quadrivalent influenza vaccines. All influenza viruses tested to date have been sensitive to the antiviral drug oseltamivir and zanamivir.

What are the implications for public health practice?

Despite less than optimal match between circulating viruses and the vaccine virus, vaccination remains the most effective method to prevent influenza and its complications. Health care providers should recommend vaccination to all unvaccinated persons aged ≥6 months now and throughout the influenza season. Treatment with influenza antiviral medications can reduce severe outcomes of influenza, when initiated as early as possible, in patients with confirmed or suspected influenza.

## Influenza-Associated Pediatric Mortality

As of December 6 (week 49), seven influenza-associated pediatric deaths that occurred in the 2014–15 season were reported to CDC. Four deaths were associated with an influenza A (H3) virus, two deaths were associated with an influenza A virus for which no subtyping was performed, and one death was associated with an influenza B virus. The number of influenza-associated pediatric deaths reported to CDC in the previous three seasons has ranged from 37 during the 2011–12 season to 171 during the 2012–13 season. During the 2009 pandemic, 358 pediatric deaths were reported from April 15, 2009, through October 2, 2010 (traditional influenza seasons include data from October [week 40] through September [week 39] of the following year).

### Discussion

As monitored by all CDC influenza surveillance systems, influenza activity in the United States for the 2014–15 season is low but increasing. Although the timing of influenza activity varies from year to year, peak activity in the United States most commonly occurs during January–March, but there can be substantial influenza activity as early as November and December. From September 28 to December 6, 2014, influenza A (H3N2) viruses were identified most frequently in the United States, but pH1N1 and influenza B viruses also were reported. Antigenic or genetic characterization of influenza-positive respiratory specimens submitted to CDC indicate that over half of the recently examined influenza A (H3N2) viruses show evidence of antigenic drift from the A/Texas/50/2012 (H3N2) virus (the H3N2 component on the 2014–15 Northern Hemisphere influenza vaccine). Even during seasons when the match between the vaccine viruses and circulating viruses is less than optimal and protection against illness might be reduced, vaccination remains the most effective method to prevent influenza and its complications. Health care providers should recommend vaccination to all unvaccinated persons aged ≥6 months now and throughout the influenza season. In 2014, the Advisory Committee on Immunization Practices recommended the preferential use of live attenuated influenza vaccine (LAIV) for healthy children aged 2 through 8 years (2). However, if LAIV is not available, inactivated influenza vaccine should be used, and vaccination should not be delayed to procure LAIV (2). Children aged 6 months through 8 years who are being vaccinated for the first time require 2 doses of influenza vaccine, administered ≥4 weeks apart (3). For children aged 6 months through 8 years who have received influenza vaccination during a previous season, health care providers should consult Advisory Committee on Immunization Practices guidelines to assess whether 1 or 2 doses are required (2).

Antiviral medications continue to be an important adjunct to vaccination for reducing the health impact of influenza. On January 21, 2011, Advisory Committee on Immunization Practices recommendations on the use of antiviral agents for treatment and chemoprophylaxis of influenza were released (4). This guidance remains in effect for the 2014–15 season, and recommended antiviral medications include oseltamivir (Tamiflu) and zanamivir (Relenza). All influenza viruses tested for the 2014–15 season since October 1 have been susceptible to oseltamivir and zanamivir. Amantadine and rimantadine are not recommended because of high levels of resistance to these drugs among circulating influenza A viruses (4). In addition, influenza B viruses are not susceptible to amantadine or rimantadine. Treatment with antivirals is recommended as soon as possible without waiting for confirmatory testing for patients with confirmed or suspected influenza who have severe, complicated, or progressive illness; who require hospitalization; or who are at higher risk for influenza complications§§§ (4). Clinical benefit is greatest when antiviral treatment is administered early. When indicated, antiviral treatment should be started as soon as possible after illness onset, ideally within 48 hours of symptom onset. However, antiviral treatment might still have some benefits in patients with severe, complicated, or progressive illness and in hospitalized patients when started after 48 hours of illness onset. Antiviral treatment also may be considered for previously healthy, symptomatic outpatients who are not considered to be at high risk and have confirmed or suspected influenza, if treatment can be initiated within 48 hours of illness onset. Residents of long-term care facilities can experience severe and fatal illness during influenza outbreaks; residents with confirmed or suspected influenza should be treated with antivirals immediately, without waiting for laboratory confirmation of influenza (4). During periods where two or more residents of long-term care facilities are ill within 72 hours with confirmed or suspected influenza, antivirals should be given prophylactically to residents and should be considered for any unvaccinated staff (4). Additionally, antiviral chemoprophylaxis can be considered for all staff, regardless of vaccination status, if the outbreak is caused by a strain of influenza virus that is not well matched to the vaccine (4).

Influenza surveillance reports for the United States are posted online weekly and are available at http://www.cdc.gov/flu/weekly. Additional information regarding influenza viruses, influenza surveillance, influenza vaccine, influenza antiviral medications, and novel influenza A virus infections in humans is available at http://www.cdc.gov/flu.

## Footnotes

* The CDC influenza surveillance system collects five categories of information from eight data sources: 1) viral surveillance (World Health Organization collaborating laboratories, the National Respiratory and Enteric Virus Surveillance System, and novel influenza A virus case reporting); 2) outpatient illness surveillance (U.S. Outpatient Influenza-Like Illness Surveillance Network); 3) mortality (122 Cities Mortality Reporting System and influenza-associated pediatric mortality reports); 4) hospitalizations (Influenza Hospitalization Surveillance Network [FluSurv-NET], which includes the Emerging Infections Program and surveillance in three additional states); and 5) summary of the geographic spread of influenza (state and territorial epidemiologist reports).

† Data reported as of December 12, 2014.

§ Region 1: Connecticut, Maine, Massachusetts, New Hampshire, Rhode Island, and Vermont. Region 2: New Jersey, New York, Puerto Rico, and the U.S. Virgin Islands. Region 3: Delaware, District of Columbia, Maryland, Pennsylvania, Virginia, and West Virginia. Region 4: Alabama, Florida, Georgia, Kentucky, Mississippi, North Carolina, South Carolina, and Tennessee. Region 5: Illinois, Indiana, Michigan, Minnesota, Ohio, and Wisconsin. Region 6: Arkansas, Louisiana, New Mexico, Oklahoma, and Texas. Region 7: Iowa, Kansas, Missouri, and Nebraska. Region 8: Colorado, Montana, North Dakota, South Dakota, Utah, and Wyoming. Region 9: Arizona, California, Hawaii, Nevada, American Samoa, Commonwealth of the Northern Mariana Islands, Federated States of Micronesia, Guam, Marshall Islands, and Republic of Palau. Region 10: Alaska, Idaho, Oregon, and Washington.

¶ CDC routinely uses hemagglutination inhibition (HI) assays to antigenically characterize influenza viruses year-round to compare how similar currently circulating influenza viruses are to those included in the influenza vaccine, and to monitor for changes in circulating influenza viruses (http://www.cdc.gov/flu/professionals/laboratory/antigenic.htm). However, a portion of recent influenza A (H3N2) viruses do not grow to sufficient hemagglutination titers for antigenic characterization by HI assays. For many of these viruses, CDC is also performing genetic characterization to infer antigenic properties.

** Defined as a temperature ≥100°F (≥37.8°C), oral or equivalent, and cough and/or sore throat, without a known cause other than influenza.

†† The national and regional baselines are the mean percentage of visits for ILI during noninfluenza weeks for the previous three seasons plus two standard deviations. A noninfluenza week is defined as periods of ≥2 consecutive weeks in which each week accounted for <2% of the season’s total number of specimens that tested positive for influenza. National and regional percentages of patient visits for ILI are weighted on the basis of state population. Use of the national baseline for regional data is not appropriate.

§§ Activity levels are based on the percentage of outpatient visits in a jurisdiction attributed to ILI and are compared with the average percentage of ILI visits that occur during weeks with little or no influenza virus circulation. Activity levels range from minimal, which would correspond to ILI activity from outpatient clinics being at or below the average, to high, which would correspond to ILI activity from outpatient clinics being much higher than the average. Because the clinical definition of ILI is very nonspecific, not all ILI is caused by influenza; however, when combined with laboratory data, the information on ILI activity provides a clearer picture of influenza activity in the United States.

¶¶ Levels of activity are 1) no activity; 2) sporadic: isolated laboratory-confirmed influenza case(s) or a laboratory-confirmed outbreak in one institution, with no increase in activity; 3) local: increased ILI, or at least two institutional outbreaks (ILI or laboratory-confirmed influenza) in one region of the state, with recent laboratory evidence of influenza in that region and virus activity no greater than sporadic in other regions; 4) regional: increased ILI activity or institutional outbreaks (ILI or laboratory-confirmed influenza) in at least two but less than half of the regions in the state with recent laboratory evidence of influenza in those regions; and 5) widespread: increased ILI activity or institutional outbreaks (ILI or laboratory-confirmed influenza) in at least half the regions in the state, with recent laboratory evidence of influenza in the state.

*** FluSurv-NET conducts population-based surveillance for laboratory-confirmed influenza-associated hospitalizations among children aged <18 years (since the 2003–04 influenza season) and adults aged ≥18 years (since the 2005–06 influenza season). FluSurv-NET covers approximately 70 counties in the 10 Emerging Infections Program states (California, Colorado, Connecticut, Georgia, Maryland, Minnesota, New Mexico, New York, Oregon, and Tennessee) and additional Influenza Hospitalization Surveillance Project (IHSP) states. IHSP began during the 2009–10 season to enhance surveillance during the 2009 H1N1 pandemic. IHSP sites included Iowa, Idaho, Michigan, Oklahoma, and South Dakota during the 2009–10 season; Idaho, Michigan, Ohio, Oklahoma, Rhode Island, and Utah during the 2010–11 season; Michigan, Ohio, Rhode Island, and Utah during the 2011–12 season; Iowa, Michigan, Ohio, Rhode Island, and Utah during the 2012–13 season; and Michigan, Ohio, and Utah during the 2013–14 and 2014–15 seasons. Incidence rates are calculated using CDC’s National Center for Health Statistics population estimates for the counties included in the surveillance catchment area. Laboratory confirmation is dependent on clinician-ordered influenza testing, and testing for influenza often is underutilized because of the poor reliability of rapid test results and greater reliance on clinical diagnosis for influenza. As a consequence, the number of cases identified as part of influenza hospitalization surveillance likely is an underestimate of the actual number of persons hospitalized with influenza.

††† The seasonal baseline proportion of P&I deaths is projected using a robust regression procedure, in which a periodic regression model is applied to the observed percentage of deaths from P&I that were reported by the 122 Cities Mortality Reporting System during the preceding 5 years. The epidemic threshold is set at 1.645 standard deviations above the seasonal baseline.

§§§ Persons at higher risk include 1) children aged <2 years; 2) adults aged ≥65 years; 3) persons with chronic pulmonary conditions (including asthma); cardiovascular disease (except hypertension alone); renal, hepatic, hematologic (including sickle cell) disease; metabolic disorders (including diabetes mellitus); or neurologic and neurodevelopmental conditions (including disorders of the brain, spinal cord, peripheral nerve, and muscle, such as cerebral palsy, epilepsy [seizure disorders], stroke, intellectual disability [mental retardation], moderate to severe developmental delay, muscular dystrophy, or spinal cord injury); 4) persons with immunosuppression, including that caused by medications or by human immunodeficiency virus infection; 5) women who are pregnant or postpartum (within 2 weeks after delivery); 6) persons aged ≤18 years who are receiving long-term aspirin therapy; 7) American Indians/Alaska Natives; 8) persons who are morbidly obese (i.e., body mass index ≥40); and 9) residents of nursing homes and other chronic care facilities.
